# Paratyphoid Fever

**Published:** 1907-10-05

**Authors:** 


### Points in Diagnosis.

### PARATYPHOID FEVER.

It is only of recent years that the possibility of there being more than one variety of typhoid fever has been discussed, and, as the result of investigations upon the subject, pathologists have drawn distinctions between ordinary typhoid fever on the one hand and paratyphoid fever on the other.

The clinician naturally asks, What are the points of distinction between the two? The briefest answer to this question is that there are no bedside differences between them; it is only in the bacteriological laboratory that they differ from each other. This being so, it might be argued that the subject was of no particular interest to the medical practitioner—that the matter was of purely scientific importance; but this, as will be seen presently, is not strictly true. Whether paratyphoid fever is a distinct disease, or only a variety of typhoid fever, is still open to discussion; but each is diagnosed with greatest certainty by Widal's clumping reaction, and it is in connection with the latter that paratyphoid fever is of importance to all doctors.

Some aspects of Widal's reaction have already been discussed in these columns, and there is no need to repeat what has already been said. There are, however, very great differences both in the rate at which the serum develops its clumping power during the fever, and also in the maximum strength attained by the Widal's reaction in different patients.

The variability in the disease, from very mild to very virulent, depends only in part upon the resisting power of the patient. Variations in virulence cannot be called differences in kind, however, and it is not upon the basis of virulence that bacteriologists distinguish paratyphoid from typhoid fever.

The *bacillus typhosus* is only distinguishable from the *bacillus coli communis* by cultural methods in skilled hands; and it has long been held that there are many different organisms loosely classed together as belonging to the "coli group." Researches have already led to the discovery of definite cultural distinctions between the *bacillus typhosus* and the *bacillus coli communis* on the one hand and certain closely allied bacilli on the other; amongst the latter are *bacillus paratyphosus A.* and *bacillus paratyphosus B.* Both kinds of paratyphoid bacilli can cause severe illness in man, clinically similar to that caused by bacillus typhosus; the similarity includes the temperature chart, the course, the abdominal condition, the spots, the enlarged spleen, the headache; it even includes the ulceration of Peyer's patches in the ileum. It will be asked, If the two diseases are so much alike in all these respects, what is the use of troubling about distinguishing them? Why not call them both typhoid fever?

The reason is this, Widal's reaction is specific. This is to say, the serum of a patient suffering from an illness caused by the *bacillus typhosus* will only clump cultures of *bacillus typhosus*; it will not clump cultures of *bacillus paratyphosus*. Similarly, the serum of a patient suffering from an illness caused by *bacillus paratyphosus A.* will only clump cultures of *bacillus paratyphosus A.*; it will not clump those of *bacillus typhosus*, and so on. The clinical importance of this will become obvious if one imagines for a moment that one has under one's care a patient suffering from a prolonged fever of obscure nature, really due to the *bacillus paratyphosus*; the diagnosis of typhoid fever will probably suggest itself, and a few drops of blood will be taken in a Widal tube and sent to the laboratory to be tested for the typhoid fever reaction. If the Widal's test is made with cultures of *bacillus typhosus* only, the report will come back "Widal's reaction negative." Perhaps it will be suggested that it is too early in the illness for it to be positive; the test may be repeated in a week's time, and again the report may be negative. Typhoid fever may thereby seem to have been excluded, and some other diagnosis, such as fungating endocarditis or pelvic inflammation, may be suggested; or the cause of the pyrexia may remain a complete mystery. The case is, to most intents and purposes, one of typhoid fever all the time; but the serum has not been tested against the proper strain of typhoid bacilli.

Paratyphoid fever, therefore, has more than a purely scientific significance; its treatment and its course are precisely similar to those of typhoid fever proper, and it is very possible that it is only a variety of the latter; but in connection with diagnosis by means of Widal's test—the most conclusive test of typhoid fever there is—the fact that there are several different "strains" of typhoid bacilli must be remembered. When a case seems to be one of typhoid fever and yet gives a negative Widal's test, it is important to know with what variety of typhoid bacillus culture the test has been made; and in repeating the test in a doubtful case various strains should be used, including the particular varieties which are recognised as the cause of paratyphoid fever.

